# The utility of patient reported data in a gynecologic oncology clinic

**DOI:** 10.1186/s40661-018-0062-4

**Published:** 2018-07-12

**Authors:** D. Barnes, R. Rivera, S. Gibson, C. Craig, J. Cragun, B. Monk, D. Chase

**Affiliations:** 1 Department of Gynecologic Oncology, Creighton University School of Medicine at St. Joseph’s Hospital and Medical Center, Phoenix, AZ USA; 2 University of Arizona Cancer Center, Tucson, AZ USA

## Abstract

**Background:**

Measuring QoL is essential to the field of gynecologic oncology but there seems to be limited standardized data regarding collecting QoL assessments throughout a patient’s cancer treatment especially in non-clinical trial patients. The aim of this study is to explore patient characteristics that may be associated with poor quality of life (QoL) in women with gynecologic cancers at two University of Arizona Cancer Center (UACC) sites.

**Methods:**

A cross-sectional survey was conducted among English speaking women with gynecologic malignancies at the University of Arizona Cancer Centers in Phoenix and Tucson from April 2012 to July 2015. The survey was a paper packet of questions that was distributed to cancer patients at the time of their clinic visit. The packet contained questions on demographic information, treatment, lifestyle characteristics, pelvic pain and Health-related quality of life (HRQoL). Measures included the generic and cancer-specific scores on the Functional Assessment of Cancer Therapy–General (FACT-G) and the Female Genitourinary Pain Index (GUPI). The total scores and subdomains were compared with descriptive variables (age, body mass index (BMI), diet, exercise, disease status, treatment and support group attendance) using Cronbach alpha (α), Spearman rank correlations (ρ), and Holm’s Bonferroni method.

**Results:**

One–hundred and forty-nine women completed the survey; 55% (*N* = 81) were older than 60 years, 38% (*N* = 45) were obese (BMI > 30), 46% (*N* = 66) exercised daily, and 84% (*N* = 111) ate one or more daily serving of fruit and vegetables. Women in remission, those who exercised daily and ate fruits/vegetables were less likely to have their symptoms impact their QoL. Younger women were more likely to report genitourinary issues (*p* = − 0.22) and overall problems with QoL (*p* = − 0.29) than older women. Among FACT-G support group responses, we found those that did not attend support groups had a significantly higher emotional wellbeing (*p* = 0.05).

**Conclusions:**

This study identified potential areas of clinical focus, which aid in understanding our approach to caring for gynecologic cancer patients and improvement of their HRQoL. We identified that age, pelvic pain, and lifestyle characteristics have indicators to poor QoL in women with gynecologic cancers. In this population, younger women and those with pelvic pain complaints, poor diet and exercise habits should be targeted early for supportive care interventions to improve QoL throughout both treatment and survivorship.

## Background

The impact of illness and treatment on quality of life (QoL) has received increasing recognition in recent years with the both the National Cancer Institute and the Food and Drug Administration who have mandated that the goals of cancer research should be to improve both survival and QoL [1]. Measuring QoL is essential to the field of gynecologic oncology but there seems to be limited standardized data regarding collecting QoL assessments throughout a patient’s cancer treatment especially in non-clinical trial patients [2, 3]. Prior studies have demonstrated that a patient’s QoL changes over the course of treatment; however this is unknown in non-clinical trial patients [4, 5].

QoL is defined as the level of satisfaction a person has with their physical (PWB), emotional (EWB), and social wellbeing (SWB) [6]. The diagnosis of cancer in a woman encompasses not only the physical effects of the disease but also the short and long-term side effects of treatment, its cost, potential economic loss, and the reaction of family and friends, each of which influence QoL [6–12]. Since QoL is a multidimensional concept it is important to assess how it affects various communities and populations so that interventions can be designed to help improve overall wellbeing in patients. Numerous instruments have been created and aim to measure patients’ QoL [13]. One of these measures is the Health Related Quality of Life (HRQoL), which allows patients to self-report symptoms using patient-reported outcome (PRO) measures [4, 14]. The use of PRO, especially in oncology, has been shown to help with detection of problematic symptoms, symptom monitoring, satisfaction with patient care, and communication between clinicians and patients [15–17].

The aim of this study is to examine HRQoL in a community oncology setting. The goal of this research is to ultimately identify and then address areas to target QoL interventions in a non-clinical trial population.

## Methods

### Study design

Following Institutional Review Board approval, we performed a cross-sectional HRQoL survey among women aged 21–89 years with gynecologic malignancies (cervical, ovarian, and/or uterine) seen at the University of Arizona Cancer Center locations in Phoenix and Tucson from April 2012 to July 2015.

### Patient selection

Participants were comprised of women who had survived cancer and were undergoing care at our two sites. Eligibility criteria included: 1) age ≥ 21 years, 2) current gynecologic malignancy 3) current history of gynecologic malignancy 4) ability to read, write, and understand English (as a primary or secondary language). Women were excluded if they did not complete at least 50% of the questionnaire.

#### QoL assessments and instruments

We used a questionnaire packet that included the Functional Assessment of Cancer Therapy Female Genitourinary Pain Index, and self-reported demographic information. The QoL questionnaires were scored separately.

*Functional Assessment of Cancer Therapy – General (FACT-G)*, is a 27-item QoL instrument that consisted of four well-being subscales: physical, functional, social, and emotional [18, 19]. Within each subdomain, questions are answered on a 5-point Likert scale, ranging from 1 (not at all) to 4 (very much). The items are summed to give a score for each subdomain. The subdomain scores are then summed to give a total FACT score; higher subscale and total scores indicate better QoL [20].

*The Genitourinary Pain Index* (*GUPI*) is a 15-item instrument intended to measure, within the past week, the intensity of three constructs: (a) pelvic pain or discomfort, (b) urinary symptoms, and (c) quality of life [21]. Lower subscale and total scores indicate better QoL.

Construct A: Pelvic pain or discomfort was measured by ten items: eight of these items, which consisted of binary response options (0 = no, 1 = yes), were indicative of pain/discomfort stemming both from the pelvic area (e.g., urethra, vagina) and activities involving the pelvic area (e.g., sexual intercourse, urinating). The ninth item measured pelvic pain frequency with six-point response options ranging from 0 (never) to 5 (always), and the tenth item used an 11-point average pain scale ranging from 0 to 10. The scores were summed to create a total score that could range from 0 to 23.

Construct B: Urinary symptoms (e.g., urinating frequency) were measured with two GUPI items with response options that ranged from 0 (not at all) to 5 (almost always). The mean of the two items were computed creating a score ranging from 0 to 5 with higher scores being indicative of higher urinary symptoms.

Construct C: GUPI- QoL consisted of three items that measured the impact of the symptoms on decreasing respondent’s QoL. The first two items have response options ranging from 0 (none) to 3 (a lot). The third item, which had response scale ranging from 1 (pleased) to 6 (terrible), measured how respondents felt about symptoms if they had them for the rest of their life. The three items were summed to create a total score, which could range from 1 to 12, with higher values being indicative of worse impact on QoL.

Self-reported demographic information such as age, weight, height, BMI, disease status (current disease verses cancer remission), cancer stage, past medical history, past surgical history, previous cancer treatment, and current chemotherapy treatment cycle was collected via self-reported questionnaire. The survey also included questions on exercise frequency (defined as ≥30 min of moderate activity), amount of daily consumption of fruits of vegetables, and support group attendance.

### Data collection

Potential study participants were approached after registering for their appointment at a clinic visit, and the objective of the study was explained. If they choose to participate, they were given the questionnaire, and patients self-reported their answers. Informed consent was obtained and presumed when patients proceeded with the questionnaire. The self-administered survey took approximately 15–20 min to complete.

### Study measures

#### Socio-demographic

Descriptive statistics were calculated for demographic and clinical characteristics. Differences in demographic, clinical, and symptoms characteristics, as well as QoL outcomes, were evaluated using Cronbach alpha (α), Spearman rank correlations (ρ), and Holm’s Bonferroni method were used to correct for type-I error rates.

### Statistical analysis

For each lifestyle behavior a one-way analysis of covariance was used to examine the association with HRQoL. Descriptive statistics and frequency distributions were performed while controlling for potential demographic and medical confounders for each lifestyle behavior. Logistic regression was then used for analysis with significance set at *p* < 0.05.

## Results

A total of 149 women participated in the study. Of the completed questionnaires 100% of patients completed the questions for age, 80% completed BMI and disease status, 88% completed diet questions, 97% completed questions for exercise, history of surgery, and support group attendance, 44% completed treatment questions, and 77% completed questions on their disease status.

Baseline self-reported demographics for the final analytic cohort are reported in Table 1. A little over half (55.4%, *N* = 81) of the women were older than 60 years, and over two-thirds (68.6%, N = 81) self-reported they were overweight or obese. About 46% reported exercising daily (*N* = 66) and 31% (*N* = 45) exercised weekly. Roughly 84% (*N* = 111) had more than one daily serving of fruit and vegetables. Only 13% (*N* = 19) of the women attended a support group.

**Table 1:** Demographic Characteristics of Participant from April 2012 to July 2015 (*N* = 149)

		*F*	%
Age (years)	21–40	15	10.07
	41–50	19	12.75
	51–60	34	22.82
	61–70	50	33.56
	> 70	31	20.81
	Total	149	100
BMI Categories (kg/m^2^)	Underweight (< 18.5)	0	0.00
	Healthy (18.5–24.99)	37	31.36
	Overweight (25–29.99)	36	30.51
	Obese (30+)	45	38.14
	Total	118	100
Exercise	Never	34	23.45
	Weekly	45	31.03
	Daily	66	45.52
	Total	145	100
Daily Amount of Fruit/ Vegetables (servings)	< 1	21	16.03
	1–2	68	51.91
	≥3	42	32.06
	Total	131	100
Attends Support Group	Yes	19	13.10
	No	126	86.90
	Total	145	100

Treatment, cancer stage, and disease status of the final analytic cohort are presented in Table 2. Prior treatment was defined as chemotherapy and/or surgery, and there were no documented treatments available for 23 individuals. Of the participants with documented treatment, 61 (48%) had a history of receiving chemotherapy, while another 61 (48%) were currently receiving chemotherapy. Only five (4%) were re-initiating chemotherapy. Among the 61 women who had a previous chemotherapy, 56% had their last treatment less than a year prior to study participation. Of the 61 patients who were currently receiving chemotherapy, 82% were in the middle (cycles 2–5) of their treatment cycle. The majority (83%, *N* = 120) of participants had either a recent or a historical report of surgery. Disease status was obtained from the questionnaires for 119 women and a little more than a quarter (27%, *N* = 32) had received their first chemotherapy dose, almost a third (33%, *N* = 39) had a recurrence of cancer, and 40% (*N* = 48) were in remission. Among participants with a documented cancer stage (*N* = 114), 60% had stage III or IV disease.

**Table 2:** Frequency and Percent Distribution of Historical/Current Treatment and Disease Progression from April 2012 to July 2015

		*F*	%
Last Previous Treatment	< 1 month	13	19.70
	2–6 month	11	16.67
	6–12	13	19.70
	13–24	7	10.61
	> 2 years	22	33.33
	Total^a^	66	100
Chemotherapy Cycle of Current Treatment	First	3	4.55
	Middle	54	81.82
	Last	9	13.64
	Total^a^	66	100
History / Recent Surgery	Yes	120	83.33
	No	24	16.67
	Total	144	100
Disease Status	1st Chemotherapy Treatment	32	26.89
	Recurrent	39	32.77
	Remission	48	40.34
	Total	119	100
Cancer Stage	I	25	21.93
	II	20	17.54
	III	43	37.72
	IV	26	22.81
	Total	114	100

^a^127 participants had a record either of only a previous treatment (*n* = 66), only a current chemotherapy treatment (*n* = 66), and for 23 cases the treatment status was not known

Table 3 summarizes the Spearman’s rho rank-order correlations (ρ) of three GUPI subscales, as well as four FACT-G dimensions, with age, BMI, healthy behaviors, surgery, and disease status. Pelvic pain or discomfort was not correlated to patient’s age, BMI, healthy behaviors (exercise engagement, daily consumption of vegetables/fruit, and group therapy attendance), or disease status (1st chemo-therapy, cancer recurrence and remission). Urinary symptoms were inversely related with age (ρ = −.22, *p* < .05) with younger patients being more likely to report urinating more frequently than older patients. BMI, exercise, history of or recent surgery, and remission had clinically significant pain severity (*p <* .05). Although the results suggest that urinary symptoms may correlate with cancer recurrence (ρ = .22, ns), the correlation was not significant.

**Table 3:** Spearman’s Rho (ρ) Rank Correlations between Patient characteristics and pain, quality of life, social support and Wellbeing from April 2012 to July 2015

		GUPI Symptoms			FACT-G Well-Being			
Patient Characteristics		Pelvic Pain	Urinary symptoms	Impact on QoL	Physical Well-Being	Social Well-Being	Emotional Well-Being	Functional Well-Being
Age	Ρ	0.09	−.22^a^	−.29^a^	.31^a^	.23^a^	0.09	.23^a^
	*N*	135	131	134	146	145	142	141
BMI	Ρ	0.04	−0.13	0.03	0.04	0.04	−0.04	0.09
	*N*	120	116	119	121	121	118	117
Exercise	Ρ	0.05	−0.03	−.20a	.22^a^	0.15	0.02	.23^a^
	*N*	130	127	129	141	140	137	136
Daily Fruit/ Vegetables	Ρ	0.09	0.04	−.23^c^	.22^a^	0.02	0.06	0.17
	*N*	127	123	126	127	127	124	124
Support Group	Ρ	0.08	−0.08	−0.01	−0.04	−0.16	−0.18	−0.03
	*N*	134	130	133	145	144	141	140
History/Recent Surgery	Ρ	0.05	0.10	.25^a^	−.24^a^	−0.03	0.04	−0.17
	*N*	133	129	132	144	143	140	139
1st Chemo	Ρ	0.13	−0.05	.22a	−.27^a^	−0.02	0.05	−0.17
	*N*	108	106	107	119	118	116	115
Recurrent Cancer	Ρ	0.08	0.22	0.16	−.23^a^	−0.09	−0.19	−.24^a^
	*N*	108	106	107	119	118	116	115
Remission	Ρ	0.04	−0.16	−.35^a^	.46^a^	0.10	0.14	.38^a^
	*N*	108	106	107	119	118	116	115
	*N*	135	131	134	146	145	142	141
	*M*	8.54	1.46	4.39	20.88	22.84	18.51	19.69
	*SD*	4.85	1.38	3.44	6.29	5.70	4.35	6.22
	*Mdn*	9.00	1.00	4.00	23.00	24.50	19.00	20.00

^a^Significant at.05 level after correcting for type I error rate with Holm–Bonferroni method

Table 3 shows that poorer QoL scores correlated with having both surgery (ρ = 0.25, *p* < 0.05) and first chemotherapy treatment (ρ = 0.22, *p <* 0.05). Women who were older were less likely to have symptoms negatively impact their QoL than younger women (ρ = − 0.29, *p* < 0.05). The more servings of fruits/vegetables (ρ = − 0.23, *p* < 0.05), a woman consumed each day or the more frequently she engaged in exercise (ρ = − 0.20, *p* < 0.05), the less likely she reported symptoms that negatively impacted her QoL. Those who were in remission were less likely to have their symptoms that affected their QoL (ρ = − 0.35, *p <* 0.05).

Participants were more likely to have higher PWB scores and fewer complaints about fatigue if they were older (ρ = 0.31, *p* < 0.05), engaged in exercise (ρ = 0.22, *p* < 0.05), had daily intake of fruits/vegetables (ρ = 0.22, *p* < 0.05), or were in remission (ρ = 0.46, *p <* 0.05). Having surgery (ρ = − 0.24, *p <* 0.05), first chemotherapy (ρ = − 0.27, *p <* 0.05), or recurrent cancer (ρ = − 0.23, *p* < .05) resulted in lower PWB. Support from friends and family as well as SWB score were positively correlated with age (ρ = 0.23). Older patients were more likely to have an increased sense of friends or family support than those who were younger. FWB had a positive association with age (ρ = 0.23, *p <* 0.05), exercise (ρ = 0.23, *p <* 0.05), and being in remission (ρ = 0.38, *p <* 0.05). FWB was negatively correlated with cancer recurrence and positively correlated with exercise. Older patients had higher FWB scores than younger patients, and those who were in remission or did not have recurrence of cancer were more functional than their counterparts.

## Discussion

Based on the survey results, we identified that age, pelvic pain, and lifestyle characteristics are indicators to poor HRQoL in women with gynecologic cancers. Previous studies have shown promise when investigating the influence of lifestyle on QoL in gynecologic cancers [22–25]. Lifestyle modifications have been shown to relieve fatigue, improve treatment induced anemia, maintain a healthy BMI, and enhance quality of sleep [26–28]. Our findings support this and indicate that weekly exercise (≥30 min of moderate activity) and a diet rich in fruits/vegetables (≥3 servings per day) positively impacted HRQoL. Almost a quarter of our population did not partake in any physical activity. This is drastically different from previous research in breast and prostate cancer survivors who reported at least > 50% of physical activity [22, 29–32]. Inferences may be that our population and the studies characteristics were different. In those studies some patients were from higher socio-economic and education backgrounds, which may be different from our patient cohort, and it would be prudent to investigate these barriers in future studies.

Alarming, however, is the finding that 44% of our younger patients ≤60 years of age reported having more issues with QoL. This is consistent with previous research among breast cancer and chronic disease patients. Younger patients have more issues adapting to their condition and have significant impairments in QoL, wellbeing, and recovery [13, 33–39]. Our data reinforces what other studies have found in chronic disease (Alzheimer’s, Multiple Sclerosis, Bone, Breast, and Non-Small Cell Lung Cancer) and in gynecologic cancers such as Ovarian Cancer; that all patients especially those younger need to be targeted and counseled early in their care about the potential side effects in their disease and treatment [36, 40–47]. This has been shown to help patients have a more realistic expectation for their outcome, decrease patient anxiety, depression, and anticipate challenges that may lie ahead during and after their treatment. Multiple studies have shown that counseling patients on chemotherapy improved QoL and physiological and physical health scores when performed by a physician [17, 48].

Another principal finding was more then half of our sample reported clinically significant pelvic pain. BMI, exercise, history of or recent surgery, and remission had clinically significant pain. Pelvic pain is a distressing symptom and a common QoL concerns for women with gynecologic cancer, and may be an influential variable of QoL in Gynecologic malignancies [47, 48]. Our data supports that pelvic pain is a direct correlate of HRQoL.

This study is not without limitations. We conducted a cross-sectional convenience sample; therefore, our results may not be generalizable, especially since only two institutions were involved within this study. By limiting our respondents to women who only spoke English as a primary or secondary language, we may have excluded a portion of our population where cultural or language factors could have influenced our results. Although we sampled patients with different disease severity (first treatment, recurrent, or remission), selection bias is still possible, but given the nature of QoL concerns, radical difference across populations seems unlikely. We did not correlate symptoms based on specific gynecologic cancers and age, and no associations were made to see how the quality or location of pelvic pain varied within the study cohort. Finally, we have not yet investigated the responsiveness of this packet and its ability to detect baseline or important changes over time, even if that change is small.

## Conclusions

This study identified potential areas of clinical focus, which aid in understanding our approach to caring for gynecologic cancer patients and improvement of their HRQoL. We identified that age, pelvic pain, and lifestyle characteristics have indicators to poor QoL in women with gynecologic cancers. In this population, younger women and those with pelvic pain complaints, poor diet and exercise habits should be targeted early for supportive care interventions to improve QoL throughout both treatment and survivorship.

### Highlights

- Lifestyle interventions may be targeted towards specific populations to improve QoL in women with gynecologic cancers
- Further studies are needed to evaluate these effects on Health Related Quality of Life (HRQoL) in non-clinical trial patient population

## Abbreviations

EMB: Emotional Wellbeing
FACT-G: Functional Assessment of Cancer Therapy–General
FWB: Functional Wellbeing
GUPI: Female Genitourinary Pain Index
HRQoL: Health-related quality of life
PRO: Patient Reported Outcome
PWB: Physical Wellbeing
QoL: Quality of Life
SWB: Social Wellbeing
